# Lights and Shadows of Long COVID: Are Latent Infections the Real Hidden Enemy?

**DOI:** 10.3390/jcm13237124

**Published:** 2024-11-25

**Authors:** Francesca Serapide, Marisa Talarico, Salvatore Rotundo, Vittorio Pascale, Riccardo Serraino, Enrico Maria Trecarichi, Alessandro Russo

**Affiliations:** 1 Dipartimento di Scienze Mediche e Chirurgiche, Università “Magna Graecia”, 88100 Catanzaro, Italy; f.serapide@unicz.it (F.S.); srotundo91@gmail.com (S.R.); r.serraino1@gmail.com (R.S.); em.trecarichi@unicz.it (E.M.T.); 2 Unità Operativa di Cardiologia, Azienda Ospedaliero Universitaria Renato Dulbecco, 88100 Catanzaro, Italy; marisa.talarico.2704@gmail.com (M.T.); vittoriopascale1@gmail.com (V.P.); 3 Unità Operativa Complessa di Malattie Infettive e Tropicali, Azienda Ospedaliera Universitaria Renato Dulbecco, 88100 Catanzaro, Italy

**Keywords:** COVID-19, Long COVID, SARS-CoV-2, post-acute sequalae, EBV, VZV, HIV, latent viral infections

## Abstract

Long COVID-19 (LC) is a poorly understood, multifactorial condition that persists for at least three months following SARS-CoV-2 infection. The underlying pathophysiological mechanisms responsible for the wide range of associated symptoms—including fatigue, brain fog, and respiratory issues—remain unclear. However, emerging evidence suggests that the reactivation of latent viral infections, such as Epstein-Barr virus, cytomegalovirus, and varicella-zoster virus, may significantly contribute to the complexity of LC. These latent viruses can be reactivated by SARS-CoV-2, contributing to a chronic inflammatory state that prolongs symptomatology. This review confirms the potential involvement of latent viral infections in LC and examines whether these infections play an independent role or act synergistically with other factors. In addition, recent studies have highlighted viral persistence and immune dysregulation as key elements in LC. Our findings suggest that preventative strategies, including vaccination and antiviral treatments during the acute phase of COVID-19, show potential in reducing LC risk by preventing viral reactivation. However, tailored diagnostic and therapeutic strategies targeting these latent infections are urgently needed. Identifying biomarkers of viral reactivation, particularly for high-risk populations, could be considered another effective strategy to mitigate LC severity. Further research is crucial to better understand the interactions between SARS-CoV-2 and latent infections, and to improve the prevention and treatment of LC.

## 1. Introduction

Long COVID-19 (LC) is a complex, multifactorial condition affecting millions worldwide. It is characterized by the persistence of symptoms unexplained by other diseases, continuing for at least three months after the onset of acute infection. The pattern of symptoms is variable.

The pathophysiological mechanisms responsible for these symptoms have yet to be clearly defined. However, the potential role of latent viral infections in the intricate web of LC-related mechanisms has gained increasing attention. Particular interest has focused on the interactions between EBV, CMV, VZV, HHV-6/7, and HIV, and their contribution to LC symptomatology.

It remains unclear whether these latent infections play an independent role in LC or if they synergistically induce a chronic inflammatory state responsible for symptomatology.

In this review, we aim to assess the role of latent viral infections in the development of LC and propose hypotheses that could guide future studies, ultimately leading to targeted diagnostic and therapeutic strategies to improve the quality of life for affected patients—a critical need in a field that remains poorly understood.

For this review, we conducted a narrative analysis of the literature using Pubmed database, including reviews, meta-analyses, original articles, and systematic reviews from the past 10 years.

## 2. Long COVID 

### 2.1. Definitions

Severe Acute Respiratory Syndrome CoronaVirus-2 (SARS-CoV-2) represents one of the most significant pandemics of the last millennium due to its rapid global spread and capacity to acquire mutations. During the pandemic, COVID-19 was associated with approximately 7 million deaths and a hospitalization rate of up to 20% [1,2], often involving severe complications [3].

The pandemic was declared over in May 2023, but the SARS-CoV-2 virus continues to circulate. As of September 2024, over 7 million individuals have been affected globally [3], although hospitalizations and related complications have significantly decreased, particularly among those excluding elderly, frail, and comorbid patients. Among these vulnerable groups, the risk of complications and hospitalization has remained high since 2021, when 3.97 million deaths were recorded [3]. 

Overall, the virus continues to circulate steadily, albeit with a lower lethality. However, the sharp reduction in vaccination campaigns has led to a significant drop in vaccination coverage. This results in an increased risk of complications for the frail population, particularly those who cannot be vaccinated or for whom the vaccine has not induced an adequate immune response [4]. 

While no specific therapy has been established against SARS-CoV-2, various therapies have shown potential protective effects, influencing disease severity. Among these, direct oral anticoagulants have been noted for cardiovascular patients [5], hormonal replacement therapy for post-menopausal women [6,7], and statins in patients with pre-existing cardiovascular conditions [8,9]. 

Recovery from COVID-19 is often complete. However, adverse clinical events may occur, even among healthy individuals. This is increasingly recognized as post-COVID-19 or LC [10], a usually disabling condition that gained widespread recognition only recently. The incidence of LC is currently estimated to be between 11% [11] and 20% [12], with up to 65 million people affected by LC as of 2024 [13]. LC is characterized by symptoms persisting for three months or more following acute COVID-19, with symptoms and signs lasting at least two months without other identifiable causes [14]. LC is responsible for significant morbidity, consequences ranging from mild to life-threatening, and significantly impacts everyday functioning. Therefore, the World Health Organization (WHO) defines LC as a condition characterized by symptoms that affect everyday life, after SARS-CoV-2 infection [15]. The latest LC definition, dated 2024, according to NASEM (National Academies of Sciences) is a chronic condition that occurs after SARS-CoV-2 infection, present for at least three months, that can affect more than one organ system, and has a significant impact on the patient’s life [16]. Notably, given that it encompasses over 200 symptoms, its specificity may be limited. This definition does not require documentation of previous COVID or necessitate that LC be a diagnosis of exclusion. Given that it includes over 200 symptoms, its specificity is limited. In the end, LC represents a pressing public health issue for at least three reasons: firstly, its incidence is alarmingly high; secondly, there is a lack of effective preventive or therapeutic interventions; and thirdly, the symptoms of LC are notably persistent. A meta-analysis involving 1.2 million individuals with symptomatic SARS-CoV-2 infection found that 15.1% (95% UI, 10.3–21.1%) experienced symptoms of LC persisting even 12 months after initial infection [17].

### 2.2. The Broad Spectrum of Long COVID Symptoms 

**Figure 1:**
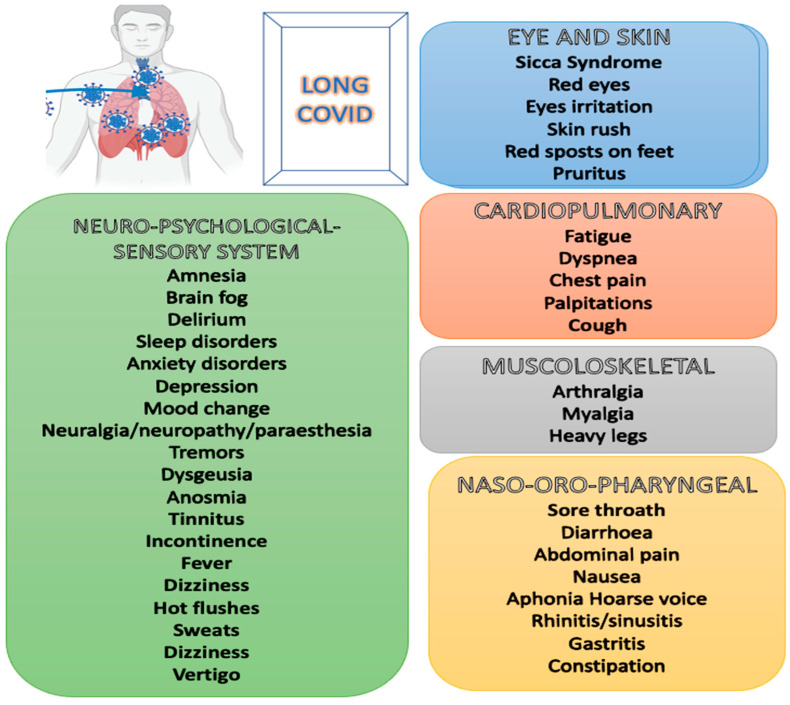
Long COVID symptoms clusters.

The spectrum of LC symptoms is wide and varies in severity. It is well-established that LC significantly impairs quality of life. More than 200 symptoms have been described to date [12] (Figure 1). These symptoms predominantly affect cardiopulmonary, naso-oropharyngeal, musculoskeletal, and neuro-psychological systems. Therefore, the most common clinical manifestations may include fatigue, mental clouding, sleep–wake rhythm alterations, arthralgia, myalgia, pharyngitis, fever, headache, gastrointestinal symptoms, rash, depression, and anxiety [18]. Another review highlighted dyspnea, cough, joint and chest pain, altered smell and taste, and diarrhea, in addition to fatigue, myalgia, and headache, as some of the most common symptoms of LC [19]. 

In a Dutch study involving 4231 patients with a history of COVID-19, matched with 8462 control participants, findings indicated that symptoms such as chest pain, difficulty breathing, pain on exhalation, muscle pain, ageusia or anosmia, general fatigue, and other autonomic dysregulation were more common among post-COVID patients (21.4%) compared to controls (8.7%). These core symptoms were also found to have increased to at least moderate severity between 90–150 days after COVID-19 diagnosis [20]. 

In a systematic review of 2100 studies, among 250,351 COVID survivors more than half of them experienced LC 6 months after recovery. Mobility impairments, pulmonary abnormalities, and mental health disorders were the most common long-term effects [21]. 

Among the LC symptoms there are various mental health conditions, and, among them, the impaired cognition called ‘brain fog’ [22]. Psychiatric disorders, even major depressive episodes, are among the most prevalent symptoms in LC [23,24] and attention must be paid in this setting to adequately treat them. 

Moreover, one-third of the population had general symptoms that were not included in any cluster, the most frequently reported were cognitive and autonomic dysfunctions frequently affect LC patients [25] chronic fatigue, post-exertional malaise [26,27], altered smell, altered taste, and diarrhea [28]. 

Di Gennaro et al., in a systematic review of 120,970 people found that the incidence of any LC symptom was 56.9% among them neurological symptoms were 19.7% (95% CI 17.4–22.1), especially difficulty in concentration (14.6%), headache, anosmia, memory deficit and cramps (over 10%). Respiratory disorders were present in a quarter of LC participants (101,849 participants, 24.5%), particularly dyspnea (24.1%) and poor exercise tolerance (16.6%). Cardiac diseases affected 11% of participants, the majority presented palpitations (11.2%) [29]. 

In summary, the most frequently complained symptoms were, according to a recent meta-analysis, fatigue, and weakness, followed by dyspnea, difficulty in doing the same daily activities, taste and/or smell dysfunction, depression, muscular and/or joint pain, affected sleep, anxiety, cough, and headache [30].

### 2.3. Identified Risk Factors for Long COVID

The relationship between LC symptoms and the severity of acute COVID-19 infection remains a topic of debate. On one hand, more symptomatic patients (five symptoms or more in the first week of infection) were found more susceptible to LC, irrespective of age and gender [31], and in mild–moderate COVID, LC symptoms seem to be more common [32]. In other studies, the persistence of symptoms was found to be independent of the initial severity of COVID [31]. In summary, LC affects survivors of COVID-19 at all disease severity, and this is one of the most controversial features. Hospitalized patients with severe COVID-19 in the acute phase seem to be at higher risk of symptoms persistence [33,34]. In a 452-patient cohort who received intensive care unit (ICU) treatment for COVID-19, one year later, 74.3% reported the presence of physical symptoms, with weakness being the most common at 38.9%. Additionally, 26.2% reported psychological symptoms, and 16.2% cognitive symptoms [35]. Even in children, hospitalization during acute phase [36] was correlated with an increased risk of developing LC. This risk was influenced by several factors such as the SARS-CoV-2 variant (Alpha and original strains), the presence of comorbidities, and age, with children aged 5–11 years and those over 12 years being particularly at risk [36]. 

Since LC affects patients irrespective of the severity of the initial illness, the aim of the current literature is to identify a high-risk profile to assess prompt intervention or, at least, more attention. In literature, there is compelling evidence linking age, female gender, smoking, and pre-existing comorbidities (asthma, obstructive pulmonary disease, untreated sleep apnea, diabetes mellitus, connective tissue disorders, obesity) [29]. Moreover, hospital admission during acute COVID-19 [37,38], the need for oxygen therapy, symptom burden (including dyspnea at presentation and chest pain), abnormal auscultation findings, and the presence of comorbidities such as asthma [28] were also linked to an increased risk of developing LC. 

A study based on 4182 patients showed that 5 symptoms during the acute phase predicted significant LC in both sexes: fatigue, headache, dyspnea, hoarse voice, and myalgia, and in patients over 70 years old, the loss of smell. Regarding pre-existing comorbidities asthma was confirmed as a ‘risk factor’, while obesity, cardiovascular diseases, and diabetes seemed to be excluded [39]. Conversely, many works underlined that cardio-vascular diseases, firstly hypertension followed by diabetes, smoking, chronic cardiovascular or lung disease, and chronic kidney failure are strong predictors of LC [40,41].

- *Age and gender*

A meta-analysis of 54 studies involving 1.2 million patients from 22 countries revealed a notable gender and age gap. It was found that LC was more common in women aged 20 years or older (10.6%) compared to men of the same age group (5.4%) three months after symptomatic SARS-CoV-2 infection [17]. Accordingly, a review by Koc et al., underlined that females are five times more likely to be at risk for LC than men [42]. Moreover, LC general, neurological, and cardiovascular symptoms were reported more frequently in females than in males [29]. While female sex is reasonably a risk factor for LC, the impact of age remains a topic of debate. In a cohort of 558 patients, people aged over 70 years (22%) showed a greater risk of developing LC ongoing symptoms lasting 4 weeks or more compared to patients aged 18–49 years (10%) [39]. By contrast, in a meta-analysis, older age was also associated with a lower risk of LC, with those aged 30–39 years having a 6% lower risk and those aged more than 70 years having a 25% lower risk compared to those aged 18–30 years. In summary, LC risk was found to follow a gradient of decreasing age [28,43].

- *Smoking*

Daily and former cigarette smoking was associated with LC among survivors [44]. Furthermore, a strong association between smoking and LC, particularly concerning respiratory, cardiovascular, and cognitive symptoms was found [45]. This association may be attributed to mechanisms involving immune dysregulation [46] or the underlying inflammatory status [47]. Moreover, smoking may prolong the recovery period for manifestations of LC [48]. Consequently, the data suggest that smoking cessation is advisable for LC prevention.

- *Obstructive sleep apnea and obstructive pulmonary disease asthma*

In a US cohort of 121,379 COPD, current daily and former smokers showed higher odds of LC [44]. In a cohort of 24,803 American patients affected by obstructive sleep apnea, its association with LC was demonstrated, especially in the untreated patients [49]. A multicenter Japanese study involving 1066 patients identified asthma and the need for mechanical ventilation as independent risk factors for prolonged dyspnea at both 6- and 12-months post-COVID-19 [50]. Among pulmonary diseases, pre-existing asthma or rhinitis have been discussed as potential LC risk factors [51,52]. However, a recent meta-analysis of 13 studies involving 9967 patients concluded that the exposure of individuals with pre-existing asthma or rhinitis to LC varied significantly, with proportions ranging from 11% to 90% [53].

- *Autoimmune conditions*

The relationship between autoimmune disorders and LC is bidirectional. In the autoimmune population, a study of 1615 patients found a 29.8% prevalence of LC, with neurological/psychological symptoms reported by 83.1% of the affected individuals. Additionally, 84% of those with LC reported an impact on their quality of life [54]. Conversely, COVID-19 was associated with an increased risk of developing autoimmune and autoinflammatory connective tissue disorders [55].

- *Obesity*

Obesity is a well-known risk factor for COVID-19 severity [56], maybe due to chronic inflammation, impaired lung function, and obesity-related conditions, which are also risk factors for COVID-19 [57]. Even if LC pathogenesis is not well-known, it was described as a SARS-CoV-2 driven brown adipose tissue dysfunction [58], of crucial importance since adipose tissue is an important SARS-CoV-2 reservoir [59]. The SARS-CoV-2 affected adipose tissue may augment systemic inflammation dysregulating its pro-inflammatory chemokines (VPF, VEGF, etc.) production, with subsequent prolonged recovery after acute infection [60]. Moreover, BMI was also associated with an increased risk of persistent symptoms. In a huge meta-analysis, individuals with a BMI greater than 30 kg/m^2^ showed a 10% higher LC risk compared to those with a BMI ranging between 18.5–25 kg/m^2^ [28].

- *Diabetes and cardiovascular diseases*

Even if a case-control study in a cohort of hospitalized COVID-19 patients (145 diabetic patients vs. 290 controls) did not find a clear correlation between pre-existing diabetes and LC [61], a meta-analysis of 18 studies including 259,978 patients showed that patients with diabetes had a significant risk of LC [62]. Even patients affected by ischemic heart disease showed a 1.28 times higher LC risk according to a meta-analysis of five studies including 201,906 patients [62].

- *Pediatric population*

In this population, the risk factors associated with LC overlap with those in the general adult population, as is moreover evidenced by a systematic review of 16 observational studies age, allergic rhinitis, obesity, previous respiratory disease, female sex, asthma, comorbidities, cardiac disease, hospitalization, and severe forms of COVID-19 are likely to be associated with an increased risk of LC [63].

- *HIV-positive population*

It was found that HIV patients carried a higher LC risk with respect to the general healthy population, because the dysfunction of the immune system would result in a higher risk of viral persistence and altered response to infection. Analyzing a sample of 39,405 HIV and COVID-19 patients, about 52% developed at least one of LC symptoms. Results from the random-effects model showed that HIV infection was associated with an increased risk of LC (odds ratio 2.20; 95% confidence interval 1.25–3.86) [64]. 

## 3. Pathophysiological Mechanisms

COVID-19 could lead to the persistence of symptoms even after the end of acute infection, as has already been demonstrated for SARS-CoV-1 and MERS-CoV.

**Figure 2:**
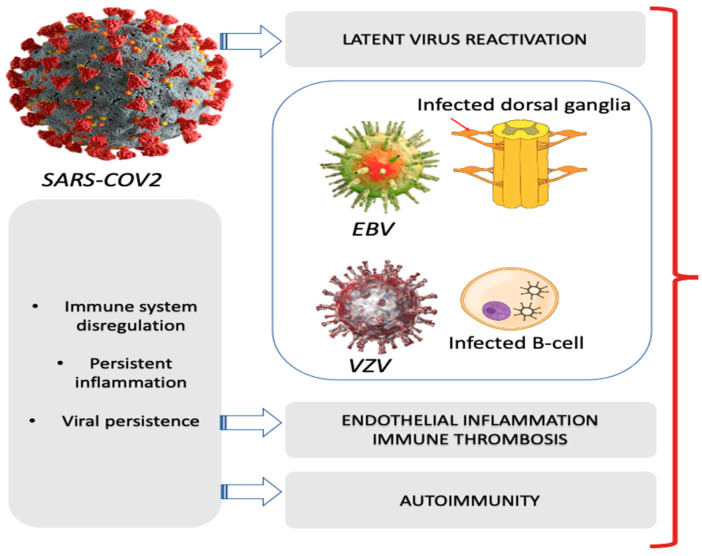
Summary of the probable pathophysiological mechanisms.

Although it has been possible to define the risk factors associated with LC, the pathophysiological mechanisms underlying this syndrome are still not well understood. Understanding them would play a crucial role in the prevention, treatment, or regression of this condition. There is not enough data in the literature to clearly define the pathological basis of the disease. The available studies have evaluated and identified the probable pathophysiological mechanisms, but none have been able to demonstrate the underlying mechanisms of this syndrome, as illustrated in Figure 2. 

One of the most accepted theories hypothesizes that this syndrome may be related to immune dysregulation leading to viral persistence, altered microbiota, autoimmunity, endothelins [65] metabolic dysregulation, and post-intensive syndrome. Specifically, elevated levels of cytokines were found in these patients compared to healthy controls, which would be associated with the persistence of symptoms [66].

In another study, 19 systematic reviews and 46 primary studies were analyzed instead, which overall showed that pathophysiological mechanisms with strong evidence were immune system dysregulation, cerebral hypoperfusion, and altered pulmonary gas exchange. Other mechanisms with moderate evidence were endothelial damage and hypercoagulation, mast cell activation, and vascular receptor autoimmunity [67].

More recent hypotheses view LC as a multifactorial disease in which several underlying mechanisms are recognized, including viral persistence, reactivation of latent virus, immune dysregulation secondary to a chronic inflammatory state, endothelial inflammation, and immune thrombosis; of lesser importance would be alterations in the gut microbiome, multiple tissue damage, and self-immunity [68].

### 3.1. Endothelial Inflammation and Immune Thrombosis

The inflammatory state induced by acute SARS-CoV-2 infection may result in a persistent state of hypercoagulability and subsequent risk of thrombotic events. This risk appears to be directly proportional to the severity of the acute infection. 

At present, the mechanisms are not very clear, but this condition may be related to inflammation-induced endothelial damage [69] as well as to the formation of autoantibodies with prothrombotic activity, including those against fibrinogen.

Indeed, vascular endothelial cells are known to express ACE2, but it remains controversial how they can support viral replication; however, direct infection of the endothelium or the indirect effects of the inflammatory cascade could lead to systemic endothelial inflammation with all its consequences.

A recent study shows that out of a sample of 113 patients with LC, 40 had typical symptoms and analysis of serum samples showed persistence, at 6-month follow-up of serological markers of tissue damage and signs of thromboinflammation, characterized by markers of endothelial activation, such as von Willebrand factor (vWF), and red blood cell lysis. These patients also showed evidence of antibody-mediated activation of the classical complement pathway, which was associated with elevated levels of anti-CMV (cytomegalovirus, also known as human herpesvirus 5) and anti-EBV (Epstein-Barr virus) immunoglobulin G (IgG) antibodies [70]. 

### 3.2. Dysregulated Immune Response and Autoimmunity

Another mechanism hypothesized to trigger LC is the chronic inflammatory state [71]. This would lead to a depletion of immune cells and chronic tissue damage, resulting in the persistence of symptoms long after the initial infection [72].

This could explain the shorter duration of LC in patients treated with dexamethasone in the acute phase, as observed in a study by Badenes Bonet et al. Cortisone would act early by modulating the inflammatory state, thereby reducing the risk of immune dysregulation secondary to a persistent inflammatory state and thereby predicting chronic tissue damage [73].

Importantly, however, studies on this topic are highly controversial and conflicting.

### 3.3. Viral Persistence and Reactivation of Latent Infections

However, viral persistence appears to be the most robust hypothesis for LC [74,75] as the virus, after an initial acute phase, would localize in a latent form in certain target organs, considered as “sanctuaries”, and through replication would lead to a chronic inflammatory state and direct cellular damage.

Evidence suggests a link between levels of viral activity during the early acute phase of infection and risk of LC [76].

Several studies have attempted to identify the main sites of viral localization in a “chronic” phase of infection. 

One of the most recent theories identifies the intestine as one of the potential “sanctuaries’’ where the virus is preserved. Indeed, intestinal biopsies have shown that there is a concentration of viral proteins in these tissues, including nucleocapsid and spike proteins, in specific regions of the intestine. However, it remains unclear which cells are predominantly involved and how they may facilitate viral persistence and replication even long after initial infection [77].

Another possible site of viral persistence is the lung. One study demonstrated the presence of SARS-CoV-2 RNA in 80 percent of lung samples collected [78].

Based on these assumptions, several studies have shown that prevention by vaccines [79,80] or treatment of acute infection with antivirals show a protective effect against LC.

A recent systematic review identified 517 studies evaluating a total sample of 2683 hospitalized and 307,409 non-hospitalized patients. This review shows that treatment of the acute phase of the disease with either remdesivir or nirmatrevir/ritonavir has a protective effect on LC. This suggests that viral persistence and thus possible latency of the virus in some “sanctuaries” may be responsible, at least in part, for the symptomatology of the LC syndrome [79].

Therefore, the best prevention strategy for LC seems to be the prevention of acute infection, although sometimes the vaccine is not able to prevent the latter, it has still been shown to be able to alleviate LC-related symptoms [81].

## 4. Long COVID Pathway: Consequence or Co-Protagonist of Reactivation of Latent and Chronic Infections?

Of particular interest is the role of some latent infections in the scenario of the mechanisms underlying LC; in fact, it is not well understood whether these infections are a direct consequence of SARS-CoV-2 infection or whether they cooperate in the pathophysiological process responsible for the symptomatic makeup of LC. 

Latent infections are characterized by their ability to camouflage themselves in host cells in a non-replicative form due to the suppressive action of the immune system. However, conditions of direct or indirect immunosuppression promote reactivation of dormant viruses, resulting in activation of viral replication and manifestation of the disease in an active form.

According to some hypotheses, the state of immune dysregulation and activation of the interferon cascade induced by acute SARS-CoV-2 infection can be considered as the main trigger for the reactivation of some herpesviruses (EBV, CMV, HHV-6, VZV, HSV) [10,18,82,83,84], which would be responsible, at least in part, for the symptomatic parade of LC. 

A recent review has analyzed the possible relationship between latent viral reactivation and the complex pathways underlying LC.

- *Epstein-Barr virus (EBV)*: A ubiquitous virus acquired by approximately 90% of the population at least once during life and may be asymptomatic if acquired during childhood. In the young adult, the clinical manifestation is characterized by marked asthenia, general malaise, pharyngo-tonsillitis, fever, headache, generalized lymphadenopathy, splenomegaly, abdominal pain, nausea and inappetence (some overlapping with the symptomatic parade of LC) [85]. Diagnosis of infection is based on clinical and microbiologic criteria. The most specific diagnostic test is the detection of specific antibodies (IgM and IgG anti-VCA, IgG anti-EBNA). The presence of anti-EBNA levels excludes acute primary infection [86]. Simultaneous activation of VCA IgM and EBNA-1 IgG indicates reactivation of latent EBV infection. After resolution of the acute phase, EBV can persist indefinitely in the host in latent form, particularly in B cells without self-manifestation. However, following conditions of immunodepression, the virus may reactivate in lytic form, leading to damage and cell death and sometimes presenting in a clinically overt form with symptoms like those of LC [85]. The pathophysiological hypotheses that EBV induces the damage responsible for the characteristic symptoms of LC are controversial and require further confirmatory studies. However, several mechanisms secondary to a chronic inflammatory state induced by previous SARS-CoV-2 infection that would promote the transition from the latent state to the lytic phase of EBV are possible. A first hypothesis suggests that the virus in the lytic phase amplifies the inflammatory state already induced by SARS-CoV-2 and contributes to the damage [87]. Another hypothesis suggests that EBV cooperates in the genesis of cellular damage with direct action on target cells [88]. The final hypothesis is that EBV plays a role in inducing immune dysregulation by triggering autoreactivity and contributing to the pathogenesis of autoimmune diseases (e.g., multiple sclerosis) [87]. To evaluate the possible interaction of the virus with the pathophysiological mechanisms of LC, the most typical approach is to identify the antibody response to EBV. Available studies have used different approaches to identify viral reactivation. One study found a potential correlation between the presence of LC symptoms, such as fatigue and neurocognitive symptoms at 4 months after initial diagnosis, which were independently associated with serologic evidence of recent EBV reactivation in the absence of viral DNA [89]. Other studies have shown a correlation between the presence of an immune response to EBV and other herpesviruses, but the role of these pathogens has not yet been elucidated [90,91]. A potential role for EBV in the mechanisms responsible for multiple sclerosis has recently been hypothesized; it appears that EBNAs may show molecular mimicry with host proteins that induce autoimmune responses [92,93,94]. Although there is insufficient evidence in the literature to confirm these hypotheses, they are of great importance in terms of prevention and treatment. Indeed, one possible strategy could be the administration of antivirals at the time of reactivation to reduce the damage associated with it. Unfortunately, no effective treatments are currently available, although some, such as acyclovir, valacyclovir and high-dose ganciclovir, have shown efficacy in vitro but not in vivo [95]. It would be necessary to intensify research in this direction to optimize the parade of symptoms associated with LC.
- *Cytomegalovirus (CMV)*: The study of the association between CMV and LC was particularly surprising. In fact, CMV seems to act as a protective factor against the occurrence of neurocognitive symptoms in LC. There is insufficient evidence in the literature, and what is available is controversial. A recent study analyzed the effect of seropositivity for CMV and LC symptoms and showed that in this category of patients there was a lower risk of developing neurocognitive symptoms of LC (Odds Ratio (OR) = 0.52, *p* = 0.036). In addition, there was no evidence of an association with other non-neurocognitive symptoms [89].

The interplay between seroconversion to CMV and LC pathways remains poorly understood. It is possible that individuals with CMV seroconversion develop a more robust immune response to SARS-CoV-2. Alternatively, it is possible that CMV-induced immunoregulatory pathways, including interleukin-10 (IL-10) secretion, may dampen local inflammation in areas of CMV reactivation, thereby reducing the risk of autoimmunity responsible for the neurological symptoms of LC [96].

- *Human Immunodeficiency Virus (HIV)*: The situation is different in HIV-positive patients. It is well known that HIV-infected people have a higher risk of developing LC symptoms than the general population, probably due to disease-related immune dysfunction. In a study of 39,405 HIV-positive patients with COVID-19, 52% of them developed at least one LC symptom. This confirms and supports the hypothesis that immune dysfunction leading to immunodeficiency or altered inflammatory pathways may promote viral persistence by inducing the onset of symptoms such as mental clouding and neurocognitive symptoms. One study also showed that a high prevalence of EBV antibodies was observed in these patients compared to patients who did not have them (51.9% vs. 32.1%, respectively, *p* < 0.01) [89], motivating the possible correlation with symptoms such as fatigue.
- *Varicella Zoster Virus (VZV):* Virus responsible for chickenpox; after initial infection, this virus remains in a latent form in the nerve ganglia and, following predisposing conditions such as immunodepression or other infections, can reactivate and manifest itself in an active form with the appearance of intensely painful and itchy vesicles. Clinical symptoms are self-limiting; however, neurological symptoms such as neuritis, neuropathy, and, in rare cases, encephalopathy and meningitis may occur. Recently, an association between LC and VZV reactivation has been observed. This hypothesis would explain the occurrence of vesicular lesions and neuropathic manifestations that worsen the clinical picture of LC patients [97].

## 5. What Strategies Are in Place?

Based on this evidence, although still unconfirmed, it is therefore possible to intervene early in the complex processes that lead to LC. It is important to pay special attention to frail patients, in whom the development of LC could complicate the very delicate balance that characterizes them. In particular, it is necessary to carry out studies that reveal the underlying pathological mechanisms in order to target diagnostic and therapeutic pathways, paying special attention to certain categories of patients who are most at risk. 

Because of the knowledge currently available, several approaches can be envisaged, but are not yet supported by robust evidence 

### 5.1. Prevention

Prevention aims to intervene before disease manifestation. It aims to identify early markers of reactivation of infection in order to prevent disease progression. 

Another possibility lies in Herpes zoster vaccine, now indicated for a limited cohort of patients, in fact it would play a role in preventing LC symptoms. 

One strategy in this regard would be to implement serologic testing protocols for latent viruses, particularly in frail patients with severe and prolonged symptoms. Serologic testing for EBV, VZV, CMV, and other herpesviruses would allow early detection of reactivation of latent infections and early intervention. The primary goal would be to identify early markers of viral reactivation (e.g., antibodies to an EBV antigen).

### 5.2. Therapeutic Strategies

Although there is currently no proven efficacy of antivirals against herpesvirus reactivation, as has been widely discussed, there is a need to conduct clinical trials aimed at identifying drugs that interact with the mechanisms that induce viral reactivation, thereby favoring a reduction in the symptomatology and severity of LC symptoms.

Another possibility lies in the possibility of modulating the induced inflammatory state and immune dysregulation secondary to SARS-CoV-2 infection and concomitant reactivated infections to reduce induced cellular damage. 

In HIV-positive patients, it would also be desirable to consider the use of targeted antiviral therapies during the acute phase of COVID-19 to reduce the risk of developing LC, given the close association between seropositivity and LC.

In patients with evidence of VZV reactivation, treatment with acyclovir or valacyclovir is indicated to limit viral replication and prevent further complications in an already immunocompromised patient.

## 6. Discussion

LC is a prevalent and debilitating condition, independent of the severity of the initial acute infection. Despite the extensive documentation of LC symptoms [6,13,14], there remains a limited understanding of the associated risk factors and pathogenetic mechanisms, which has subsequent therapeutic implications. Regarding potential clinical risk factors, age, female gender, smoking, and pre-existing comorbidities especially asthma, obstructive pulmonary disease, untreated sleep apnea, diabetes mellitus, connective tissue disorders, obesity were described [23,24,35,36]. In addition, hospitalization during the acute phase [31,32,33] and smoking [38] have been identified as potential risk factors. However, pathogenetic mechanisms are still unknown. Current literature primarily focuses on several proposed mechanisms, including viral persistence, reactivation of latent viruses, immune dysregulation secondary to a chronic inflammatory state, endothelial inflammation, and immune thrombosis. To a lesser extent, alterations in the gut microbiome, multiple tissue damage, and autoimmunity have also been suggested as contributory factors [68]. The most intriguing hypothesis posits that acute SARS-CoV-2 infection can be considered as the main trigger for the reactivation of some herpesviruses, especially EBV, VZV, HHV-6 and HIV [75,76,77]. These viruses share numerous clinical and pathogenetic characteristics with LC. In particular, EBV, VZV, HIV, and CMV have been linked to some of the neurological, dermatological symptoms, and general malaise and fatigue observed in LC [78,79,80,81,82,83,84,85,86,87,88,89,90]. The hypothesis regarding EBV suggests that the virus could be reactivated into a lytic phase due to the chronic inflammation induced by SARS-CoV-2, or as a consequence of an autoimmune process. For this reason, to evaluate the possible interaction of the virus with the pathophysiological mechanisms of LC, available studies have used different approaches to identify viral reactivation, the most common methods are to identify the antibody response to latent viruses, especially EBV chronic inflammatory state [80,81,82,83,84,85,86,87].

## 7. Conclusions

In conclusion, COVID-19 remains a poorly understood condition with substantial consequences for the quality of life and well-being of affected individuals. This highlights the need for further research to better manage this ongoing health crisis, which continues to evolve, albeit in a less overt manner.

## 8. Future Directions

In our opinion, two aspects should be underlined. The first is to anticipate, in the setting of LC, the eventual EBV/VZV/HHV-6/HIV viral re-activation diagnosis and to identify new and specific biomarkers and risk factors of both LC and latent viral reactivation.

The second is to provide prompt, targeted treatments. Despite the current lack of proven antiviral efficacy against herpesvirus reactivation, clinical trials should focus on identifying drugs that can prevent viral reactivation and thereby mitigate LC symptoms.

Expanding the cohort of patients for whom the herpes zoster vaccine is indicated would also be appropriate.

Another possibility lies in the reduction of inflammatory state.

## Data Availability

No new data were created or analyzed in this study. Data sharing is not applicable to this article.

## References

[1] Huang C., Wang Y., Li X., Ren L., Zhao J., Hu Y., Zhang L., Fan G., Xu J., Gu X. (2020). Clinical Features of Patients Infected with 2019 Novel Coronavirus in Wuhan, China. Lancet.

[2] Richardson S., Hirsch J.S., Narasimhan M., Crawford J.M., McGinn T., Davidson K.W., Barnaby D.P., Becker L.B., Chelico J.D., the Northwell COVID-19 Research Consortium (2020). Presenting Characteristics, Comorbidities, and Outcomes Among 5700 Patients Hospitalized With COVID-19 in the New York City Area. JAMA.

[3] https://data.who.int/dashboards/covid19/cases?n=c.

[4] Rotundo S., Berardelli L., Gullì S., La Gamba V., Lionello R., Russo A., Trecarichi E.M., Torti C. (2024). Early Initiation of Combined Therapy in Severely Immunocompromised Patients with COVID-19: A Retrospective Cohort Study. BMC Infect. Dis..

[5] Rossi R., Coppi F., Talarico M., Boriani G. (2020). Protective Role of Chronic Treatment with Direct Oral Anticoagulants in Elderly Patients Affected by Interstitial Pneumonia in COVID-19 Era. Eur. J. Intern. Med..

[6] Choi S.W., Kim J., Lee J.H., Kim S.K., Lee S.R., Kim S.H., Chae H.D. (2022). Hormone Therapy in the Era of the COVID-19 Pandemic: A Review. J. Menopausal Med..

[7] Averyanova M., Vishnyakova P., Yureneva S., Yakushevskaya O., Fatkhudinov T., Elchaninov A., Sukhikh G. (2022). Sex Hormones and Immune System: Menopausal Hormone Therapy in the Context of COVID-19 Pandemic. Front. Immunol..

[8] Rossi R., Talarico M., Coppi F., Boriani G. (2020). Protective Role of Statins in COVID 19 Patients: Importance of Pharmacokinetic Characteristics Rather than Intensity of Action. Intern. Emerg. Med..

[9] Kow C.S., Hasan S.S. (2020). Meta-Analysis of Effect of Statins in Patients with COVID-19. Am. J. Cardiol..

[10] Proal A.D., VanElzakker M.B. (2021). Long COVID or Post-Acute Sequelae of COVID-19 (PASC): An Overview of Biological Factors That May Contribute to Persistent Symptoms. Front. Microbiol..

[11] Kavanagh K.T., Cormier L.E., Pontus C., Bergman A., Webley W. (2024). Long COVID’s Impact on Patients, Workers, & Society: A Review. Medicine.

[12] Parums D.V. (2024). Long COVID or Post-Acute Sequelae of SARS-CoV-2 Infection (PASC) and the Urgent Need to Identify Diagnostic Biomarkers and Risk Factors. Med. Sci. Monit..

[13] Makhluf H., Madany H., Kim K. (2024). Long COVID: Long-Term Impact of SARS-CoV2. Diagnostics.

[14] Rofail D., Somersan-Karakaya S., Choi J.Y., Przydzial K., Zhao Y., Hussein M., Norton T.D., Podolanczuk A.J., Mylonakis E., Geba G.P. (2024). Thematic Analysis to Explore Patients’ Experiences with Long COVID-19: A Conceptual Model of Symptoms and Impacts on Daily Lives. BMJ Open.

[15] Soriano J.B., Murthy S., Marshall J.C., Relan P., Diaz J. (2022). V A Clinical Case Definition of Post-COVID-19 Condition by a Delphi Consensus. Lancet Infect. Dis..

[16] Fineberg H.V., Brown L., Worku T., Goldowitz I. (2024). A Long COVID Definition.

[17] Wulf Hanson S., Abbafati C., Aerts J.G., Al-Aly Z., Ashbaugh C., Ballouz T., Blyuss O., Bobkova P., Bonsel G., Borzakova S. (2022). Estimated Global Proportions of Individuals With Persistent Fatigue, Cognitive, and Respiratory Symptom Clusters Following Symptomatic COVID-19 in 2020 and 2021. JAMA.

[18] Vojdani A., Vojdani E., Saidara E., Maes M. (2023). Persistent SARS-CoV-2 Infection, EBV, HHV-6 and Other Factors May Contribute to Inflammation and Autoimmunity in Long COVID. Viruses.

[19] Aiyegbusi O.L., Hughes S.E., Turner G., Rivera S.C., McMullan C., Chandan J.S., Haroon S., Price G., Davies E.H., Nirantharakumar K. (2021). Symptoms, Complications and Management of Long COVID: A Review. J. R. Soc. Med..

[20] Ballering A.V., van Zon S.K.R., olde Hartman T.C., Rosmalen J.G.M. (2022). Persistence of Somatic Symptoms after COVID-19 in the Netherlands: An Observational Cohort Study. Lancet.

[21] Groff D., Sun A., Ssentongo A.E., Ba D.M., Parsons N., Poudel G.R., Lekoubou A., Oh J.S., Ericson J.E., Ssentongo P. (2021). Short-Term and Long-Term Rates of Postacute Sequelae of SARS-CoV-2 Infection. JAMA Netw. Open.

[22] van der Feltz-Cornelis C., Turk F., Sweetman J., Khunti K., Gabbay M., Shepherd J., Montgomery H., Strain W.D., Lip G.Y.H., Wootton D. (2024). Prevalence of Mental Health Conditions and Brain Fog in People with Long COVID: A Systematic Review and Meta-Analysis. Gen. Hosp. Psychiatry.

[23] Taquet M., Dercon Q., Luciano S., Geddes J.R., Husain M., Harrison P.J. (2021). Incidence, Co-Occurrence, and Evolution of Long-COVID Features: A 6-Month Retrospective Cohort Study of 273,618 Survivors of COVID-19. PLoS Med..

[24] Naidu S.B., Shah A.J., Saigal A., Smith C., Brill S.E., Goldring J., Hurst J.R., Jarvis H., Lipman M., Mandal S. (2021). The High Mental Health Burden of “Long COVID” and Its Association with on-Going Physical and Respiratory Symptoms in All Adults Discharged from Hospital. Eur. Respir. J..

[25] Michelen M., Manoharan L., Elkheir N., Cheng V., Dagens A., Hastie C., O’Hara M., Suett J., Dahmash D., Bugaeva P. (2021). Characterising Long COVID: A Living Systematic Review. BMJ Glob. Health.

[26] Klein J., Wood J., Jaycox J.R., Dhodapkar R.M., Lu P., Gehlhausen J.R., Tabachnikova A., Greene K., Tabacof L., Malik A.A. (2023). Distinguishing Features of Long COVID Identified through Immune Profiling. Nature.

[27] Salari N., Khodayari Y., Hosseinian-Far A., Zarei H., Rasoulpoor S., Akbari H., Mohammadi M. (2022). Global Prevalence of Chronic Fatigue Syndrome among Long COVID-19 Patients: A Systematic Review and Meta-Analysis. Biopsychosoc. Med..

[28] Subramanian A., Nirantharakumar K., Hughes S., Myles P., Williams T., Gokhale K.M., Taverner T., Chandan J.S., Brown K., Simms-Williams N. (2022). Symptoms and Risk Factors for Long COVID in Non-Hospitalized Adults. Nat. Med..

[29] Di Gennaro F., Belati A., Tulone O., Diella L., Fiore Bavaro D., Bonica R., Genna V., Smith L., Trott M., Bruyere O. (2023). Incidence of Long COVID-19 in People with Previous SARS-Cov2 Infection: A Systematic Review and Meta-Analysis of 120,970 Patients. Intern. Emerg. Med..

[30] Lippi G., Cervellin G. (2014). Risk Assessment of Post-Infarction Heart Failure. Systematic Review on the Role of Emerging Biomarkers. Crit. Rev. Clin. Lab. Sci..

[31] Arnold D.T., Hamilton F.W., Milne A., Morley A.J., Viner J., Attwood M., Noel A., Gunning S., Hatrick J., Hamilton S. (2021). Patient Outcomes after Hospitalisation with COVID-19 and Implications for Follow-up: Results from a Prospective UK Cohort. Thorax.

[32] Carvalho-Schneider C., Laurent E., Lemaignen A., Beaufils E., Bourbao-Tournois C., Laribi S., Flament T., Ferreira-Maldent N., Bruyère F., Stefic K. (2021). Follow-up of Adults with Noncritical COVID-19 Two Months after Symptom Onset. Clin. Microbiol. Infect..

[33] Zhang H., Huang C., Gu X., Wang Y., Li X., Liu M., Wang Q., Xu J., Wang Y., Dai H. (2024). 3-year outcomes of discharged survivors of COVID-19 following the SARS-CoV-2 omicron (B.1.1.529) wave in 2022 in China: A longitudinal cohort study. Lancet Respir Med..

[34] Huang C., Huang L., Wang Y., Li X., Ren L., Gu X., Kang L., Guo L., Liu M., Zhou X. (2023). 6-Month Consequences of COVID-19 in Patients Discharged from Hospital: A Cohort Study. Lancet.

[35] Heesakkers H., van der Hoeven J.G., Corsten S., Janssen I., Ewalds E., Simons K.S., Westerhof B., Rettig T.C.D., Jacobs C., van Santen S. (2022). Clinical Outcomes Among Patients With 1-Year Survival Following Intensive Care Unit Treatment for COVID-19. JAMA.

[36] Camporesi A., Morello R., La Rocca A., Zampino G., Vezzulli F., Munblit D., Raffaelli F., Valentini P., Buonsenso D. (2024). Characteristics and Predictors of Long Covid in Children: A 3-Year Prospective Cohort Study. EClinicalMedicine.

[37] Cogliandro V., Bonfanti P. (2024). Long COVID: Lights and Shadows on the Clinical Characterization of This Emerging Pathology. New Microbiol..

[38] Jovanoski N., Chen X., Becker U., Zalocusky K., Chawla D., Tsai L., Borm M., Neighbors M., Yau V. (2021). Severity of COVID-19 and Adverse Long-Term Outcomes: A Retrospective Cohort Study Based on a US Electronic Health Record Database. BMJ Open.

[39] Sudre C.H., Murray B., Varsavsky T., Graham M.S., Penfold R.S., Bowyer R.C., Pujol J.C., Klaser K., Antonelli M., Canas L.S. (2021). Attributes and Predictors of Long COVID. Nat. Med..

[40] Chan Sui Ko A., Candellier A., Mercier M., Joseph C., Schmit J.-L., Lanoix J.-P., Andrejak C. (2022). Number of Initial Symptoms Is More Related to Long COVID-19 than Acute Severity of Infection: A Prospective Cohort of Hospitalized Patients. Int. J. Infect. Dis..

[41] Mendelson M., Nel J., Blumberg L., Madhi S.A., Dryden M., Stevens W., Venter F.W.D. (2020). Long-COVID: An Evolving Problem with an Extensive Impact. South Afr. Med. J..

[42] Koc H.C., Xiao J., Liu W., Li Y., Chen G. (2022). Long COVID and Its Management. Int. J. Biol. Sci..

[43] Yong S.J. (2021). Long COVID or post-COVID-19 syndrome: Putative pathophysiology, risk factors, and treatments. Infect. Dis..

[44] Erinoso O., Osibogun O., Balakrishnan S., Yang W. (2024). Long COVID among US Adults from a Population-Based Study: Association with Vaccination, Cigarette Smoking, and the Modifying Effect of Chronic Obstructive Pulmonary Disease (COPD). Prev. Med..

[45] Trofor A.C., Robu Popa D., Melinte O.E., Trofor L., Vicol C., Grosu-Creangă I.A., Crișan Dabija R.A., Cernomaz A.T. (2024). Looking at the Data on Smoking and Post-COVID-19 Syndrome—A Literature Review. J. Pers. Med..

[46] Syed U., Subramanian A., Wraith D.C., Lord J.M., McGee K., Ghokale K., Nirantharakumar K., Haroon S. (2023). Incidence of Immune-Mediated Inflammatory Diseases Following COVID-19: A Matched Cohort Study in UK Primary Care. BMC Med..

[47] Moro-García M.A., Echeverría A., Galán-Artímez M.C., Suárez-García F.M., Solano-Jaurrieta J.J., Avanzas-Fernández P., Díaz-Molina B., Lambert J.L., López-Larrea C., Morís de la Tassa C. (2014). Immunosenescence and Inflammation Characterize Chronic Heart Failure Patients with More Advanced Disease. Int. J. Cardiol..

[48] Takakura K., Suka M., Kajihara M., Koido S. (2023). Clinical Features, Therapeutic Outcomes, and Recovery Period of Long COVID. J. Med. Virol..

[49] Quan S.F., Weaver M.D., Czeisler M.É., Barger L.K., Booker L.A., Howard M.E., Jackson M.L., Lane R.I., McDonald C.F., Ridgers A. (2024). Association of Obstructive Sleep Apnea with Post-Acute Sequelae of SARS-CoV-2 Infection. Am. J. Med..

[50] Matsuyama E., Miyata J., Terai H., Miyazaki N., Iwasaki T., Nagashima K., Watase M., Sunata K., Namkoong H., Asakura T. (2024). Chronic Obstructive Pulmonary Disease, Asthma, and Mechanical Ventilation Are Risk Factors for Dyspnea in Patients with Long COVID: A Japanese Nationwide Cohort Study. Respir. Investig..

[51] Notarte K.I., de Oliveira M.H.S., Peligro P.J., Velasco J.V., Macaranas I., Ver A.T., Pangilinan F.C., Pastrana A., Goldrich N., Kavteladze D. (2022). Age, Sex and Previous Comorbidities as Risk Factors Not Associated with SARS-CoV-2 Infection for Long COVID-19: A Systematic Review and Meta-Analysis. J. Clin. Med..

[52] Philip K.E.J., Buttery S., Williams P., Vijayakumar B., Tonkin J., Cumella A., Renwick L., Ogden L., Quint J.K., Johnston S.L. (2022). Impact of COVID-19 on People with Asthma: A Mixed Methods Analysis from a UK Wide Survey. BMJ Open Respir. Res..

[53] Wolff D., Drewitz K.P., Ulrich A., Siegels D., Deckert S., Sprenger A.A., Kuper P.R., Schmitt J., Munblit D., Apfelbacher C. (2023). Allergic Diseases as Risk Factors for Long-COVID Symptoms: Systematic Review of Prospective Cohort Studies. Clin. Exp. Allergy.

[54] Teles M.S., Brundage J., Chiang T.P.-Y., Alejo J.L., Henriquez N., Wallwork R., Christopher-Stine L., Massie A., Segev D.L., Connolly C.M. (2024). Prevalence and Risk Factors of Postacute Sequelae of COVID-19 in Adults With Systemic Autoimmune Rheumatic Diseases. J. Rheumatol..

[55] Lim S.H., Ju H.J., Han J.H., Lee J.H., Lee W.-S., Bae J.M., Lee S. (2023). Autoimmune and Autoinflammatory Connective Tissue Disorders Following COVID-19. JAMA Netw. Open.

[56] Popkin B.M., Du S., Green W.D., Beck M.A., Algaith T., Herbst C.H., Alsukait R.F., Alluhidan M., Alazemi N., Shekar M. (2020). Individuals with Obesity and COVID-19: A Global Perspective on the Epidemiology and Biological Relationships. Obes. Rev..

[57] Stefan N., Birkenfeld A.L., Schulze M.B., Ludwig D.S. (2020). Obesity and Impaired Metabolic Health in Patients with COVID-19. Nat. Rev. Endocrinol..

[58] Reese J.T., Blau H., Casiraghi E., Bergquist T., Loomba J.J., Callahan T.J., Laraway B., Antonescu C., Coleman B., Gargano M. (2023). Generalisable Long COVID Subtypes: Findings from the NIH N3C and RECOVER Programmes. EBioMedicine.

[59] Kruglikov I.L., Scherer P.E. (2020). The Role of Adipocytes and Adipocyte-Like Cells in the Severity of COVID-19 Infections. Obesity.

[60] Favre G., Legueult K., Pradier C., Raffaelli C., Ichai C., Iannelli A., Redheuil A., Lucidarme O., Esnault V. (2021). Visceral Fat Is Associated to the Severity of COVID-19. Metabolism.

[61] Fernández-de-las-Peñas C., Guijarro C., Torres-Macho J., Velasco-Arribas M., Plaza-Canteli S., Hernández-Barrera V., Arias-Navalón J.A. (2021). Diabetes and the Risk of Long-Term Post-COVID Symptoms. Diabetes.

[62] Tsampasian V., Elghazaly H., Chattopadhyay R., Debski M., Naing T.K.P., Garg P., Clark A., Ntatsaki E., Vassiliou V.S. (2023). Risk Factors Associated with Post−COVID-19 Condition. JAMA Intern. Med..

[63] Rayner D.G., Wang E., Su C., Patel O.D., Aleluya S., Giglia A., Zhu E., Siddique M. (2023). Risk Factors for Long COVID in Children and Adolescents: A Systematic Review and Meta-Analysis. World J. Pediatr..

[64] Yang X., Shi F., Zhang H., Giang W.A., Kaur A., Chen H., Li X. (2024). Long COVID among People with HIV: A Systematic Review and Meta-analysis. HIV Med..

[65] Russo A., Serapide F., Quirino A., Tarsitano M.G., Marascio N., Serraino R., Rotundo S., Matera G., Trecarichi E.M., Torti C. (2022). Microbiological and Clinical Findings of SARS-CoV-2 Infection after 2 Years of Pandemic: From Lung to Gut Microbiota. Diagnostics.

[66] Tziolos N.-R., Ioannou P., Baliou S., Kofteridis D.P. (2023). Long COVID-19 Pathophysiology: What Do We Know So Far?. Microorganisms.

[67] Diar Bakerly N., Smith N., Darbyshire J.L., Kwon J., Bullock E., Baley S., Sivan M., Delaney B. (2024). Pathophysiological Mechanisms in Long COVID: A Mixed Method Systematic Review. Int. J. Environ. Res. Public Health.

[68] Greenhalgh T., Sivan M., Perlowski A., Nikolich J.Ž. (2024). Long COVID: A Clinical Update. Lancet.

[69] Pelle M.C., Tassone B., Ricchio M., Mazzitelli M., Davoli C., Procopio G., Cancelliere A., La Gamba V., Lio E., Matera G. (2020). Late-onset myocardial infarction and autoimmune haemolytic anaemia in a COVID-19 patient without respiratory symptoms, concomitant with a paradoxical increase in inflammatory markers: A case report. J. Med. Case Rep..

[70] Cervia-Hasler C., Brüningk S.C., Hoch T., Fan B., Muzio G., Thompson R.C., Ceglarek L., Meledin R., Westermann P., Emmenegger M. (2024). Persistent Complement Dysregulation with Signs of Thromboinflammation in Active Long Covid. Science.

[71] Russo A., Serraino R., Serapide F., Bruni A., Garofalo E., Longhini F., Trecarichi E.M., Torti C. (2024). COVID-19-Associated Pulmonary Aspergillosis in Intensive Care Unit: A Real-Life Experience. Heliyon.

[72] Evans R.A., Leavy O.C., Richardson M., Elneima O., McAuley H.J.C., Shikotra A., Singapuri A., Sereno M., Saunders R.M., Harris V.C. (2022). Clinical Characteristics with Inflammation Profiling of Long COVID and Association with 1-Year Recovery Following Hospitalisation in the UK: A Prospective Observational Study. Lancet Respir. Med..

[73] Badenes Bonet D., Caguana Vélez O.A., Duran Jordà X., Comas Serrano M., Posso Rivera M., Admetlló M., Herranz Blasco A., Cuadrado Godia E., Marco Navarro E., Martin Ezquerra G. (2023). Treatment of COVID-19 during the Acute Phase in Hospitalized Patients Decreases Post-Acute Sequelae of COVID-19. J. Clin. Med..

[74] Fernández-de-las-Peñas C., Raveendran A.V., Giordano R., Arendt-Nielsen L. (2023). Long COVID or Post-COVID-19 Condition: Past, Present and Future Research Directions. Microorganisms.

[75] Nalbandian A., Sehgal K., Gupta A., Madhavan M.V., McGroder C., Stevens J.S., Cook J.R., Nordvig A.S., Shalev D., Sehrawat T.S. (2021). Post-Acute COVID-19 Syndrome. Nat. Med..

[76] Antar A.A.R., Yu T., Demko Z.O., Hu C., Tornheim J.A., Blair P.W., Thomas D.L., Manabe Y.C. (2023). Long COVID Brain Fog and Muscle Pain Are Associated with Longer Time to Clearance of SARS-CoV-2 RNA from the Upper Respiratory Tract during Acute Infection. Front. Immunol..

[77] McMillan P., Turner A.J., Uhal B.D. (2024). Mechanisms of Gut-Related Viral Persistence in Long COVID. Viruses.

[78] Roden A.C., Boland J.M., Johnson T.F., Aubry M.C., Lo Y.-C., Butt Y.M., Maleszewski J.J., Larsen B.T., Tazelaar H.D., Khoor A. (2022). Late Complications of COVID-19. Arch. Pathol. Lab. Med..

[79] Fernández-de-las-Peñas C., Torres-Macho J., Catahay J.A., Macasaet R., Velasco J.V., Macapagal S., Caldararo M., Henry B.M., Lippi G., Franco-Moreno A. (2024). Is Antiviral Treatment at the Acute Phase of COVID-19 Effective for Decreasing the Risk of Long-COVID? A Systematic Review. Infection.

[80] Català M., Mercadé-Besora N., Kolde R., Trinh N.T.H., Roel E., Burn E., Rathod-Mistry T., Kostka K., Man W.Y., Delmestri A. (2024). The Effectiveness of COVID-19 Vaccines to Prevent Long COVID Symptoms: Staggered Cohort Study of Data from the UK, Spain, and Estonia. Lancet Respir. Med..

[81] Azzolini E., Levi R., Sarti R., Pozzi C., Mollura M., Mantovani A., Rescigno M. (2022). Association Between BNT162b2 Vaccination and Long COVID After Infections Not Requiring Hospitalization in Health Care Workers. JAMA.

[82] Li Y., Schneider A.M., Mehta A., Sade-Feldman M., Kays K.R., Gentili M., Charland N.C., Gonye A.L.K., Gushterova I., Khanna H.K. (2021). SARS-CoV-2 Viremia Is Associated with Distinct Proteomic Pathways and Predicts COVID-19 Outcomes. J. Clin. Investig..

[83] Siberry V.G.R., Rowe P.C. (2022). Pediatric Long COVID and Myalgic Encephalomyelitis/Chronic Fatigue Syndrome. Pediatr. Infect. Dis. J..

[84] Howard-Jones A.R., Burgner D.P., Crawford N.W., Goeman E., Gray P.E., Hsu P., Kuek S., McMullan B.J., Tosif S., Wurzel D. (2022). COVID-19 in Children. II: Pathogenesis, Disease Spectrum and Management. J. Paediatr. Child. Health.

[85] Huang W., Bai L., Tang H. (2023). Epstein-Barr Virus Infection: The Micro and Macro Worlds. Virol. J..

[86] Leung A.K.C., Lam J.M., Barankin B. (2024). Infectious Mononucleosis: An Updated Review. Curr. Pediatr. Rev..

[87] Peluso M.J., Deeks S.G. (2024). Mechanisms of Long COVID and the Path toward Therapeutics. Cell.

[88] Davis H.E., McCorkell L., Vogel J.M., Topol E.J. (2023). Long COVID: Major Findings, Mechanisms and Recommendations. Nat. Rev. Microbiol..

[89] Peluso M.J., Deveau T.-M., Munter S.E., Ryder D., Buck A., Beck-Engeser G., Chan F., Lu S., Goldberg S.A., Hoh R. (2023). Chronic Viral Coinfections Differentially Affect the Likelihood of Developing Long COVID. J. Clin. Investig..

[90] Shikova E., Reshkova V., Kumanova A., Raleva S., Alexandrova D., Capo N., Murovska M., on behalf of the European Network on ME/CFS (EUROMENE) (2020). Cytomegalovirus, Epstein-Barr Virus, and Human Herpesvirus-6 Infections in Patients with Myalgic Encephalomyelitis/Chronic Fatigue Syndrome. J. Med. Virol..

[91] Zhang L., Gough J., Christmas D., Mattey D.L., Richards S.C.M., Main J., Enlander D., Honeybourne D., Ayres J.G., Nutt D.J. (2010). Microbial Infections in Eight Genomic Subtypes of Chronic Fatigue Syndrome/Myalgic Encephalomyelitis. J. Clin. Pathol..

[92] Bjornevik K., Cortese M., Healy B.C., Kuhle J., Mina M.J., Leng Y., Elledge S.J., Niebuhr D.W., Scher A.I., Munger K.L. (2022). Longitudinal Analysis Reveals High Prevalence of Epstein-Barr Virus Associated with Multiple Sclerosis. Science (1979).

[93] Lanz T.V., Brewer R.C., Ho P.P., Moon J.-S., Jude K.M., Fernandez D., Fernandes R.A., Gomez A.M., Nadj G.-S., Bartley C.M. (2022). Clonally Expanded B Cells in Multiple Sclerosis Bind EBV EBNA1 and GlialCAM. Nature.

[94] Houen G., Trier N.H. (2021). Epstein-Barr Virus and Systemic Autoimmune Diseases. Front. Immunol..

[95] Yager J.E., Magaret A.S., Kuntz S.R., Selke S., Huang M.-L., Corey L., Casper C., Wald A. (2017). Valganciclovir for the Suppression of Epstein-Barr Virus Replication. J. Infect. Dis..

[96] Poole E., Neves T.C., Oliveira M.T., Sinclair J., da Silva M.C.C. (2020). Human Cytomegalovirus Interleukin 10 Homologs: Facing the Immune System. Front. Cell Infect. Microbiol..

[97] Huang S., Yeh W., Chen H., Yong S. (2023). Long-term Risk of Herpes Zoster Following COVID-19: A Retrospective Cohort Study of 2 442 686 Patients. J. Med. Virol..

